# Infective Endocarditis of the TAVI Prosthesis: Emerging New Challenges

**DOI:** 10.3390/diagnostics15070814

**Published:** 2025-03-23

**Authors:** Georgiana Pintea Bentea, Brahim Berdaoui, Ahmad Awada, Behrouz Sina, Ahmed Sanoussi

**Affiliations:** 1 Department of Cardiology, CHU Brugmann, 1020 Brussels, Belgium; 2 Department of Cardiac Surgery, CHU Brugmann, 1020 Brussels, Belgium

**Keywords:** transcatheter aortic valve implantation, infective endocarditis, diagnostic image

## Abstract

A 74-year-old patient presented to the emergency department with aggravating asthenia and persistent fever over the course of the last 2 weeks. He benefited 3 years prior from a self-expandable transcatheter aortic valve implantation (TAVI) for symptomatic severe aortic valve stenosis, as he refused open heart surgery. The blood workup showed leukocytosis and high C-reactive protein levels. However, the microbiological analysis remained negative. During his hospital stay, a transesophageal echocardiogram was performed, which showed thickening of the transcatheter heart valve leaflets and a vegetation of almost 2 cm attached to the stent of the TAVI. A high suspicion of endocarditis was established at this stage even in the absence of microbiological documentation. Surgery showed extensive damage to the prosthesis and consisted of the careful disengagement of the TAVI followed by a successful aortic valve replacement. The microbiological analysis of the excised TAVI identified *Streptococcus bovis* as the culprit bacteria. The patient fully recovered at the 1-year follow-up. TAVI endocarditis is a source of emerging new challenges, as they are becoming more frequent due to the increase in TAVI procedures, can affect the leaflets and the stent, and can present destructive evolution even with less aggressive bacteria, while remaining amenable to surgical treatment.

**Figure 1:**
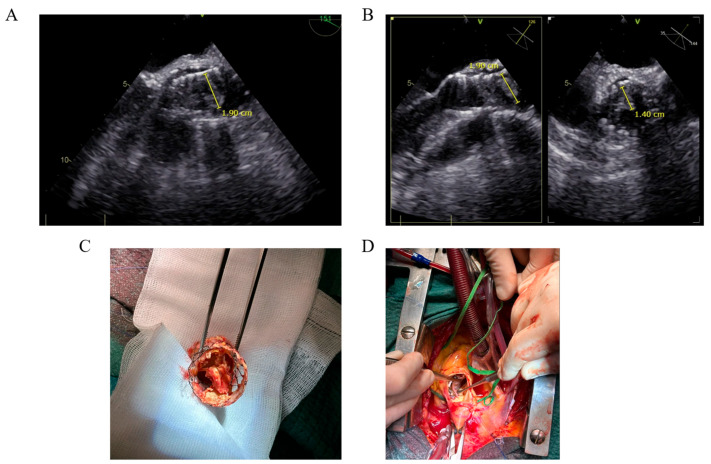
We describe the case of a 74-year-old patient who presented to the emergency department with asthenia, fever, and dyspnea that continued to worsen over the course of the last 2 weeks. The patient presented no other symptoms such as cough or gastrointestinal or urinary tract symptoms. He benefited 3 years prior from a self-expandable transcatheter aortic valve implantation (TAVI) for symptomatic severe aortic valve stenosis and triple vessel coronary stenting, as he refused open heart surgery. The patient also had paroxysmal atrial fibrillation, treated with flecainide for rhythm control and anticoagulated by apixaban. Shortly after his admission to the emergency department, the patient presented with a cardiac arrest with wide QRS complex tachycardia due to flecainide toxicity in the setting of acute kidney failure. The resuscitation maneuvers were successful and the patient recovered. Given the hemodynamic instability of the patient culminating in cardiac arrest associated with fever for the last 2 weeks, leukocytosis and high C-reactive protein, the patient was started on broad-spectrum antibiotics (Ceftriaxone) without blood cultures being performed before the first dose. In view of the recovered cardiac arrest and the substantial cardiovascular background of the patient, the cardiological team was promptly involved in the management of the patient. The transthoracic ultrasound showed moderate TAVI prosthesis dysfunction with a maximal gradient of 2.7 m/s and moderate paraprosthetic regurgitation. The transesophageal echocardiogram (TEE) showed thickening of the transcatheter heart valve leaflets and a vegetation of almost 2 cm attached to the stent of the TAVI (Panel (**A**,**B**), Supplementary Materials Videos S1 and S2). A high suspicion of endocarditis was established even in the absence of microbiological documentation; as such, Linezolid and Gentamicin were added to the antibiotic regimen. The hypothesis of a thrombus was less likely as the patient was anticoagulated by apixaban for atrial fibrillation. The patient benefited from 9 blood culture sets in the 5 days waiting for the heart surgery, all coming back negative even for the presence of a fungal infection. Furthermore, infectious serologies for atypical bacteria were performed, which proved to be negative. The multiple discussions within the Endocarditis Team suspected the implication of a Streptococcus spp. given the rapid microbiological decapitation with only one dose of Ceftriaxone and the subacute evolution of the symptoms a few weeks before admission, which contrasts with the more acute evolution of an endocarditis due to Staphylococcus or Gram-negative bacteria. Moreover, the hypothesis of a fungal endocarditis was evoked; however, it was considered less likely given that the patient was not immunocompromised. The Endocarditis Team established the surgical indication, which was performed in an urgent manner, within 5 days, according to the European Society of Cardiology (ESC) guidelines on the management of infective endocarditis [1]. The decision was based on the size of the vegetation and hence its high embolism risk and the poor hemodynamic tolerance of the dysfunctioning aortic valve prosthesis. Surgery showed extensive damage to the prosthesis (Panel (**C**)) and consisted of the careful disengagement of the TAVI (Panel (**D**)) followed by a successful aortic valve replacement using a sutureless aortic bioprosthetic valve. The culprit bacterium was identified on the excised TAVI as Streptococcus bovis by the 16S rRNA sequencing PCR technique, and a targeted antibiotherapy with Amoxicillin was started for a total duration of 6 weeks. The combined surgical and antimicrobial therapy management was successful, as the patient could leave the hospital after the prolonged intravenous course of antibiotics. At the one year follow-up, the patient had completely recovered and was doing well. The incidence of TAVI infective endocarditis is reportedly more modest than that observed in surgical prostheses [2], and the majority of cases occur within the 3 months following the implantation procedure [3], and do not present vegetations visible by TEE [4]. The particularities of this case include a late-onset infective endocarditis of a TAVI prosthesis and the presence of vegetation both on the leaflets and the stents of the TAVI readily observed in TEE. Furthermore, surprisingly in this case, the microbiological diagnosis of endocarditis was particularly challenging, as the culprit bacterium was identified on the excised TAVI as Streptococcus bovis by the 16S rRNA sequencing PCR technique. The in-hospital mortality of TAVI endocarditis is reported as high as 36%, attaining almost 60% at the one-year follow-up [5]. On the other hand, the in-hospital mortality of surgical valve endocarditis is reported at approximately 20% [6]. The ESC guidelines [1] regarding the antimicrobial therapy are the same for TAVI endocarditis and surgical valve endocarditis; however, patients with TAVI endocarditis benefit from surgery a lot less than those with surgical valve endocarditis, e.g., approximately 25% versus 45%, respectively [7]. These differences in the management and prognosis of TAVI and surgical valve endocarditis could be due to different patient populations, as patients benefiting from TAVI are older, frailer, and present with a higher burden of comorbidities, while TAVI endocarditis is more often associated with Staphylococcus aureus [5,8]. In line with this, more recent studies including younger patients in the TAVI group did not reveal differences in one-year mortality when comparing TAVI endocarditis to surgical valve endocarditis [7]. While the majority of studies to date failed to unveil the benefit of surgery in infective endocarditis associated with TAVI [8], these are non-randomized, small-sample studies. However, one study [7] did show the benefit of surgery over conservative treatment in the case of TAVI endocarditis, especially when associated with local complications. In the current case, the patient presented a very sizeable vegetation on the stent of the TAVI and damage to its leaflets, leading to a dysfunctioning aortic valve prosthesis. Furthermore, despite the multiple blood cultures analyzed, a microbiological documentation of the endocarditis was difficult to establish, and as such hindered a targeted antibiotic therapy. Consequently, a broad-spectrum therapy including 3 antibiotics was administered until the identification of the culprit bacterium on the surgically excised TAVI, each with its side effects and only assumed efficacy. The surgical option in this case proved to be beneficial in both local and systemic treatment of the TAVI endocarditis. Furthermore, the patient did not present the same burden of comorbidities and frailty as usual patients benefiting from TAVI, as he initially refused the open heart surgery. TAVI endocarditis is a source of emerging new challenges, as it is becoming more frequent due to the increase in TAVI procedures, can manifest in a variety of clinical presentations, can affect the leaflets and the stent, and can present destructive evolution even with less aggressive bacteria, while remaining amenable to surgical treatment.

## Data Availability

The data presented in this study are available on request from the corresponding author due to privacy reasons.

## Supplementary Materials

The following supporting information can be downloaded at: https://www.mdpi.com/article/10.3390/diagnostics15070814/s1, Videos S1 and S2: The transesophageal echocardiogram showed thickening of the transcatheter heart valve leaflets and a vegetation of almost 2 cm attached to the stent of the TAVI.
